# The Treatment of Autism Spectrum Disorder With Auditory Neurofeedback: A Randomized Placebo Controlled Trial Using the Mente Autism Device

**DOI:** 10.3389/fneur.2018.00537

**Published:** 2018-07-05

**Authors:** Frederick R. Carrick, Guido Pagnacco, Ahmed Hankir, Mahera Abdulrahman, Rashid Zaman, Emily R. Kalambaheti, Derek A. Barton, Paul E. Link, Elena Oggero

**Affiliations:** ^1^Neurology, Carrick Institute, Cape Canaveral, FL, United States; ^2^Bedfordshire Centre for Mental Health Research in Association with University of Cambridge, Cambridge, United Kingdom; ^3^Harvard Macy Institute and MGH Institute of Health Professions, Boston, MA, United States; ^4^Bioengineering, Carrick Institute, Cape Canaveral, FL, United States; ^5^Department of Electrical and Computer Engineering, University of Wyoming, Laramie, WY, United States; ^6^Psychiatry, Carrick Institute, Cape Canaveral, FL, United States; ^7^Leeds York Partnership NHS Foundation Trust, Leeds, United Kingdom; ^8^Department of Medical Education, Dubai Health Authority, Dubai, United Arab Emirates; ^9^Department of Primary Health Care, Dubai Medical College, Dubai, United Arab Emirates; ^10^Psychiatry, University of Cambridge, Cambridge, United Kingdom; ^11^Neurology, Plasticity Brain Center, Orlando, FL, United States

**Keywords:** autism spectrum disorder, neurofeedback, posturography, qeeg guided neurofeedback, binaural beats

## Abstract

**Introduction:** Children affected by autism spectrum disorder (ASD) often have impairment of social interaction and demonstrate difficulty with emotional communication, display of posture and facial expression, with recognized relationships between postural control mechanisms and cognitive functions. Beside standard biomedical interventions and psychopharmacological treatments, there is increasing interest in the use of alternative non-invasive treatments such as neurofeedback (NFB) that could potentially modulate brain activity resulting in behavioral modification.

**Methods:** Eighty-three ASD subjects were randomized to an Active group receiving NFB using the Mente device and a Control group using a Sham device. Both groups used the device each morning for 45 minutes over a 12 week home based trial without any other clinical interventions. Pre and Post standard ASD questionnaires, qEEG and posturography were used to measure the effectiveness of the treatment.

**Results:** Thirty-four subjects (17 Active and 17 Control) completed the study. Statistically and substantively significant changes were found in several outcome measures for subjects that received the treatment. Similar changes were not detected in the Control group.

**Conclusions:** Our results show that a short 12 week course of NFB using the Mente Autism device can lead to significant changes in brain activity (qEEG), sensorimotor behavior (posturography), and behavior (standardized questionnaires) in ASD children.

## Introduction

Autism spectrum disorder (ASD) affects approximately 1% of children in the community and as a disorder with lifetime consequences, postponing its identification and intervention beyond infancy can be considered a precious loss of time (1). Autistic children demonstrate difficulty with emotional communication, display of posture and facial expression (2). They often have impairment of social interaction, demonstrating stereotypical patterns of behavior or interests as well as communication deficits that affect both verbal and nonverbal strategies (1, 3). This is complicated by restricted interests and repetitive patterns of behavior affecting their communicative skills, attention, empathy and social activities (4). Fortunately, biomedical interventions and psychopharmacological treatments of ASD are helpful, but unfortunately they may be associated with risks and side effects (5). As a consequence, many parents and their clinicians search for alternative methods of treatment. For instance, the treatment of childhood developmental disorders (CDD) commonly includes complementary and alternative medical (CAM) interventions such as nutritional supplementation, efforts to reduce environmental toxins and biofeedback (BFB). In addition to these treatments, neurofeedback (NFB), a noninvasive BFB approach shown to enhance neuroregulation and metabolic function in ASD is proving to be efficacious (4, 6). Porges' polyvagal theory is used to emphasize the need to integrate NFB with BFB, that in turn influence dynamic brain circuitry (7, 8). Such integration combining surface electroencephalography signals emerged in the 1970s (9) and has been successful in the treatment of a variety of psychiatric disorders of childhood (10) such that these noninvasive effective interventions have become very attractive in the treatment of ASD (11). The central outcome goal of these therapies is to modulate the activity of the brain to result in a behavioral modification. We desired to ascertain the consequences of a novel NFB in the treatment of ASD using the Mente Autism (AAT Medical, Malta) active portable NFB device composed of a headset, software and a cloud component. The Mente device is novel and unique and motivated us to conduct a clinical trial to test its effectiveness in the treatment of ASD as there are no published studies in this clinical area.

The Mente device is designed to utilize the EEG activity of children diagnosed with ASD to provide a home-based support therapy in order to promote relaxation as well as engagement of the subject. The device reads the EEG, augmented in a real-time NFB training in association with auditory therapy that is delivered through binaural beat sounds transmitted via earphones connected directly to the headband. The headband houses 4-EEG channels and a bias electrode system. The EEG signal is bipolar between the 2 EEG Sensors placed on the front of the head (Fp1 and Fp2), taken as active, and each of the 2 sensors placed at the back of the head (O1 and O2), taken as reference. The fifth sensor (FPz, placed between Fp1 and Fp2) is used as bias to minimize the DC and common-mode AC signals. This system can be used as a stand-alone system, with all collected raw EEG data being stored and processed on the device, thus allowing for the user to move around freely without any hindrance from added devices.

All the analyses are performed on the Mente device with the raw data from each channel (Fp1-O1, Fp1-O2, Fp2-O1, Fp2-O2) passed through two 121th order FIR filters (High pass: Fstop1 = 0.25 Hz, Fpass1 = 1.25 Hz; Low pass: Fpass2 = 40 Hz, Fstop2 = 42 Hz), then an automated Eye Blink correction (12) is applied to remove eye blink artifacts. Then the signal is averaged between all the channels with a Fast Fourier Transformation (FFT). The following bands are selected from the FFT: Delta (1–3 Hz), Theta (4–7 Hz), Alpha (8–13 Hz), Beta1 (14–19 Hz), Beta2 (20–35 Hz). The results are updated every second and an auditory feedback is delivered in the form of binaural auditory beats. The binaural beats produce a perceptual phenomenon that occurs when two tones of a slightly different frequency are presented separately to the left and right ears resulting in the listener perceiving a single tone that varies in amplitude at a frequency equal to the frequency difference between the two tones. The binaural beats delivered by Mente Autism are in the range of delta, theta and beta frequency and are selected accordingly to the user's predominant frequency. The NFB protocol delivered by the device aims to reduce the abnormal EEG pattern associated with ASD that is characterized by excessive power at low-frequency (delta and theta) and high-frequency (beta) bands, as well as reduced power in the middle-range frequency band (alpha). At the end of a treatment session, the data collected are sent to a secure cloud system where they are stored. Subjects in the Control group used a device identical to the Mente Autism device, but the binaural beats were randomly generated and not based on the EEG pattern recorded by the device.

We needed robust outcome measurements that could tell us if the Mente NFB device was effective. We have extensive clinical experience using Quantitative electroencephalography (qEEG), a non-invasive technique that allows a highly precise measurement of brain function and connectivity and decided to incorporate it in our investigation. Additionally, qEEG can identify patterns of brain activity by the amplitude of brain waves at various locations in the cerebral cortex (7) with neural conductivity abnormalities found at both the local and global levels in ASD (13). Moreover, it can distinguish children suffering from ASD from normal children and can be used as an instrument to evaluate the efficacy of an intervention or treatment (14). The electroencephalographic data of qEEG has been utilized in neurofeedback treatments of a variety of childhood psychiatric disorders (10) and was utilized in this investigation.

To ascertain the consequences of NFB in the treatment of ASD, in this study, the effect of a 12-week long NFB treatment plan using the Mente Autism device was investigated by comparing pre and post qEEG and posturography results in children affected by ASD. Standard questionnaires pre and post were also used to better evaluate the effect of the treatment.

## Materials and methods

This investigation was approved by our Institutional Review Board (approval #20160321001) and registered with ClinicalTrials.gov maintained by the National Library of Medicine (NLM) at the National Institutes of Health (NIH) registration # NCT02773303. It was conducted according to the Declaration of Helsinki and for all the subjects in this investigation, the parents gave their written informed consent and the children gave their assent.

### Protocol

The study protocol included two evaluations, one (PRE) at enrollment and the second (POST) after the conclusion of a treatment period of 12 weeks of home-based NFB therapy. The evaluations consisted of qEEG, dynamic computerized posturography, and ASD questionnaires including the Autism Treatment Evaluation Checklist (ATEC), the Social Responsiveness Scale–Second Edition (SRS-2), the Behavior Rating Inventory of Executive Function (BRIEF), the Autism Behavior Checklist (ABC) and the Questions About Behavioral Function (QABF). Subjects were randomly assigned to two groups: Active (receiving the actual therapy) and Control (receiving a placebo/sham therapy).

### Subjects

A power and sample size calculation suggested that we needed a total of 32 subjects with 16 subjects assigned to each of the two groups to maintain an alpha < 0.05 with 80% power. Our experience as a tertiary brain center has demonstrated that there often is difficulty for patients to return for testing after 3 months, especially if a treatment was considered to be successful. We had no incentive for the subjects in this study to return for outcome testing. We expected that we might lose a large percent of our sample size and decided to randomize 84 subjects to two groups of 42 (Active and Control) in order to plan for study dropouts and obtain the desired number of subjects. Subjects were recruited by placing notices of the study on social media Autism sites.

### Inclusion-exclusion criteria

Subjects were enrolled in the study if they were 2–18 years of age and had received a previously documented diagnosis of ASD and a targeted neural profile (high delta/high theta and/or high beta) associated with autistic disorders was confirmed by qEEG evaluation. Furthermore, since the therapy was going to be administered via a device requiring to be connected to a computer, the availability of an Internet connection was necessary for all subjects. Moreover, since the study testing was done in our clinic in Orlando FL, the parents of accepted subjects agreed that they would be responsible to come to the clinic at the beginning of the study and after 12 weeks of home based therapy and that they would bear the expenses associated with the visits. Subjects were excluded if they had a history of hearing impairment and co-morbidities such as Rett-Syndrome. No healthy controls were included in this research.

#### Treatment device

The NFB protocol delivered by the Mente Autism device aims to reduce the abnormal EEG pattern associated with ASD that is characterized by excessive power at low frequency and high-frequency bands, as well as reduced power in the middle-range frequency band. As previously mentioned, Mente Autism utilizes an auditory feedback in the form of binaural auditory beats. The binaural beats produce a perceptual phenomenon that occurs when two tones of a slightly different frequency are presented separately to the left and right ears resulting in the listener perceiving a single tone that varies in amplitude at a frequency equal to the frequency difference between the two tones (15). The binaural beats delivered by Mente Autism are in the range of delta, theta and beta frequency and are selected accordingly to the user's brain pattern (amount and distribution of brain waves). Changes in the sound volume are controlled by specialized algorithms and the user receives an instant feedback through the earphones. The Mente Autism system presents a mixture of warble tones and binaural beats using a set of generative rules from derived brain activity levels, in order to present multiple frequencies that are hypothesized to alter and help the brain work in more desirable patterns. The protocol aims to promote a self-regulation of the brain activity including also the reduction of the faster high beta waves mostly associated with anxiety and over arousal. Subjects in the Control group used a device identical to the Mente Autism device, but the binaural beats were randomly generated and not based on the EEG pattern recorded by the device. An iPad running current OS and smart card Internet access was provided to all subjects at no cost, although they were advised that they could use their own devices, smart phones and tablets if they desired.

### Outcome data acquisition and analysis

#### qEEG

qEEG is produced through statistical analysis of the EEG, i.e., conversion of the time domain EEG record (voltage plotted against time) to the frequency domain (amplitude or power plotted against frequency) using the fast Fourier transformation (FFT) (16). The qEEG bands we considered are delta (1.5–3.5 Hz), theta (3.5–7.5 Hz), alpha (7.5–12.5 Hz), beta (12.5–30 Hz), and gamma (30–70 Hz) (17). Subject classifications are based on multivariate analysis of linear combinations of qEEG measures (discriminant functions, or “discriminants”), an approach called “neurometric” analysis (18, 19). In this study, raw EEG data were collected non-invasively from the participant's scalp at the time of the PRE and POST evaluation using a BrainMaster Discovery 20 channel EEG (BrainMaster Technologies; Bedford, Ohio). Electrode caps were used to place recording electrodes over the 19 standard regions defined by the International 10/20 system referenced to linked ears:Fp1, Fp2, F3, F4, F7, F8, T3, T4, C3, C4, P3, P4, T5, T6, O1, O2, Fz, Cz, and Pz. The electrode impedance levels were kept below 5,000 Ω. All channels of EEG were acquired with 24 bits resolution at the sampling rate of 256 Hz. The EEG was recorded for 5 min while the subject rested with eyes closed. Prior to the quantification analysis, all EEG signals were examined to remove epochs containing artifacts, such as EEG segments contaminated by horizontal and lateral eye-movement, muscle activity and electrocardiac artifact. Successive EEG quantification was restricted to those children from whom a minimum of 1 min of artifact-free EEG could be obtained. The NeuroGuide EEG and qEEG analysis system software (Applied Neuroscience, Inc., Largo, FL) was used for the signal processing of the qEEG. Each qEEG measure was calculated with the respect to the mean and standard deviation of that measure obtained from an age-regressed normal database using a Z or standard score. The normative database from Neuroguide provided an evaluation of whether the subject's qEEG deviated from the normative sample (20). From the qEEG, we considered the absolute power transformed into z-scores obtained by the power spectrum of each EEG channel. Multiple ANCOVAs were conducted with treatment as between subject factor, post-scores as dependent variables and pre-tests scores as covariate. Paired *t*-test were also conducted within the Active and Control groups respectively. Statistical analysis of qEEG measurements were performed using IBM SPSS Statistics release 20.0.0 (IBM Corporation, Armonk, NY, U.S.A.) Normal distribution of the data was assessed using the Kolmogorov-Smirnov (with the Lilliefors Significance Correction) and the Shapiro-Wilk tests before further proceeding with the analysis. Paired *t*-tests were applied for exploring the differences between pre- and post-test scores with regard to absolute z-scores in group and for each frequency band and the effect size was calculated as Cohen's d (21) (0.2 is considered to represent a small effect size, 0.5 represents a medium effect size and 0.8 a large effect size).

#### Posturography

The ability of a subject to maintain equilibrium was assessed using standard posturography testing in four different conditions [known as the modified Clinical Test of Sensory Integration in Balance or mCTSIB protocol (22)]: standing on a hard surface with eyes open or closed and on a compliant surface (a 0.1 m thick foam cushion) again with eyes open or closed. We used the CAPS Professional system (Vestibular Technologies, LLC – Cheyenne WY- USA), a commercially available posturography medical device registered with the FDA and proven (23) to exceed the metrological characteristics for clinical posturography recommended by the International Society for Posture and Gait Research (24). Subjects were instructed to stand upright in a comfortable position, with their arms held loosely and naturally to their sides. No restrictions were imposed on the position of the feet and subjects were instructed to stand within the boundaries of the mostly triangular area of support of the force platform (~ 0.75 m × 0.85 m) and of the foam cushion (~ 0.6 m × 0.38 m) allowing subjects to use their preferred and natural stance. Center of Pressure (CoP) coordinates were acquired at 64 Hz for 20 seconds and then upsampled to 1 kHz before any analysis was performed. No filtering besides the anti-aliasing filtering in the device was applied to the data. The 95% confidence mediolateral sway (95% Conf MLSway), the 95% confidence antero-posterior sway (95% Conf AP Sway), the 95% confidence maximum sway (95% Conf Max Sway, the largest sway in any direction), the average sway velocity (Average Sway Vel, calculated as the sway path length (how much the CoP moved during the test) divided by the duration of the test or Average Sway Vel), and the 95% confidence ellipse area (95% Conf Ellipse Area), were considered. For each subject, the different sway measurements and the average sway velocity were normalized by the subject's height, and the 95% confidence ellipse area was normalized by the square of the subject's height to remove any subject's gender and height dependency (25). The statistical analysis of the data was performed using IBM SPSS Statistics release 20.0.0 (IBM Corporation, Armonk, NY, U.S.A.).The normality of the distribution of the data was confirmed using the Kolmogorov-Smirnov (with the Lilliefors Significance Correction) and the Shapiro-Wilk tests. Since the posturography results consisted of 5 variables per test condition, rather than performing individual *t*-test comparison, the differences in the PRE and POST assessments between Active and Control groups were examined using General Linear Model (GLM) analysis, whereas the differences PRE-POST in the Active and Control groups were investigated using repeated measures GLM analysis.

### Questionnaires

Five commonly available and validated questionnaires were used pre and post treatment to determine the status of the participants: *The Autism Treatment Evaluation Checklist (ATEC)* (26), *The Social Responsiveness Scale–Second Edition (SRS-2)* (27), *The Behavior Rating Inventory of Executive Function (BRIEF)* (28)*, The Autism Behavior Checklist (ABC)* (29)*, Questions About Behavioral Function (QABF)* (30)

## Results

727 subjects expressed interest in the study and were assessed for eligibility and 644 were excluded (113 did not meet the inclusion criteria, 266 declined to participate and 265 were not chosen in the randomization procedure). 84 subjects were randomized into two groups but one subject did not show for the PRE examination resulting in 83 subjects randomized into Active (41) and Control (42) groups. All randomized subjects received the intervention for their group with the Active group receiving a real Mente Autism device and the Control group receiving a sham/placebo Mente Autism device. Of the 83 subjects that completed the evaluation at the enrollment time, 34 returned for the POST evaluation after the 12 weeks of home based therapy. The dropout reasons were inability or unwillingness to come back for the POST (22 in the Active group and 16 in the Control group), or discontinued treatment because of problems associated with internet connections and technology challenges (2 in the Active group and 9 in the Control group). No subjects dropped out because of problems of tolerance with the Mente device or treatment. Furthermore, some subjects could not complete some of the testing resulting in a different number of subjects included in the different analysis. Some children could not tolerate the placement of an EEG Cap while others could not stand still for 20 seconds of posturography testing.

**Figure 1:**
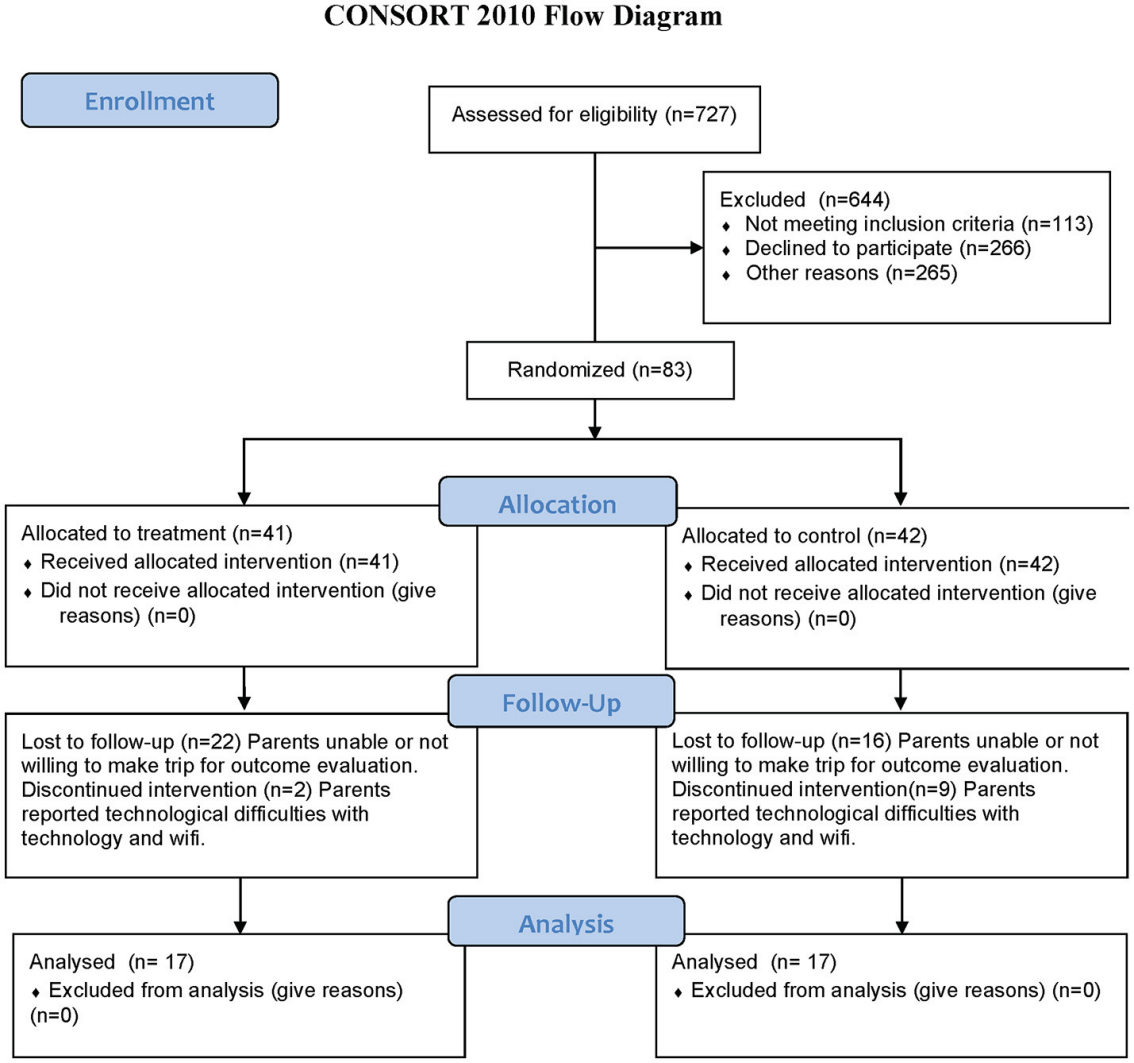
Consort flow diagram.

Figure 1 shows the Consort Flow Diagram.

Table 1 shows the demographic information for the Active group, the Control group and combined for all the subjects that completed the protocol and that were then included in the various analyzes. 34 participants completed all the assessments pre and post treatment. Because of artifacts from movement and problems with tolerating the EEG cap, 10 subjects were removed from the qEEG analysis. Therefore, from the entire sample, 24 participants were included in the successive qEEG analysis with their age expressed in months. A Body Mass Index (BMI) is also included in the table. No gender discrimination was included in the demographics of the posturographic data as once they are normalized there is no gender dependency (25).

**Table 1:** Demographics of the subjects participating in the study and that were included in the various analysis.

**Analysis**	**Demographics at enrollment**					
		**Group**	**# of subjects**	**Gender**	**Median age [year]**	**Age range [years]**
qEEG		Control	12	10 males 2 female Total	8 13 8.5	5–15 11–15 5–15
		Active	12	11 males 1 female Total	12 5 11.5	6–17 5–5 5–17
		Combined	24	21 males 3 females Total	9 11 10	5–17 5–15 5–17
	**Group**	**# of subjects**	**Age [months]**	**Height [m]**	**Mass [kg]**	**BMI [kg/m**^2^**]**
Posturography	Control	17	115.5 (9.4)	1.41 (0.05)	40.7 (4.5)	19.28 (0.99)
	Active	17	117.9 (10.0)	1.37 (0.04)	34.7 (3.5)	17.32 (0.81)
	*p*-value	—	0.858	0.509	0.251	0.133
	Combined	34	116.7 (6.8)	1.39 (0.03)	37.4 (2.9)	18.30 (0.64)
	Average value (Standard Error of the Mean) for the different parameters *T*-tests between the active and control groups calculated to determine if there were any statistically significant differences among the two groups					
		**Group**	**# of subjects**	**Gender**	**Median age [year]**	**Age range [years]**
ATEC SRS-2 ABC QABF Questionnaires		Control	17	13 males 4 females Total	7 11.5 8	4–15 11–15 4–15
		Active	17	15 males 2 females Total	11 11 11	6–16 5–17 5–17
		Combined	34	28 males 6 females Total	8 11.5 9.5	4–16 5–17 4–17
		**Group**	**# of subjects**	**Gender**	**Median age [year]**	**Age range [years]**
BRIEF Questionnaire (after excluding subjects with high Inconsistency scale)		Control	13	12 males 1 female Total	7 15 7	4–15 15–15 4–15
		Active	16	14 males 2 females Total	11 11 11	6–16 5–17 5–17
		Combined	29	26 males 3 females Total	8 15 8	4–16 5–17 4–17

*The different number of subjects in the qEEG analysis is due to the exclusion of some subjects due to artifacts in the EEG recordings. The different number of subjects in the BRIEF analysis is due to the removal of the subject with high inconsistency value*.

The BRIEF includes an “*Inconsistency scale*” that evaluates individual scores in order to monitor the response rate as a score equal or higher than 7 representing an indication of a high degree of inconsistency in the responses. We identified 5 subjects who demonstrated significant scores on the Inconsistency scale promoting a further analysis excluding these subjects. This new sample was composed of 29 subjects (and their demographics is indicated separately in Table 1). Furthermore, since they are the most complete, the demographics of the posturographic analysis were also used to determine if there were any statistically significant differences among the two groups by calculating the *t*-tests between the Active and Control groups for each demographic variable. The resulting *p*-values are also shown, indicating that no differences were present among the two groups pre-treatment.

### qEEG

**Figure 2:**
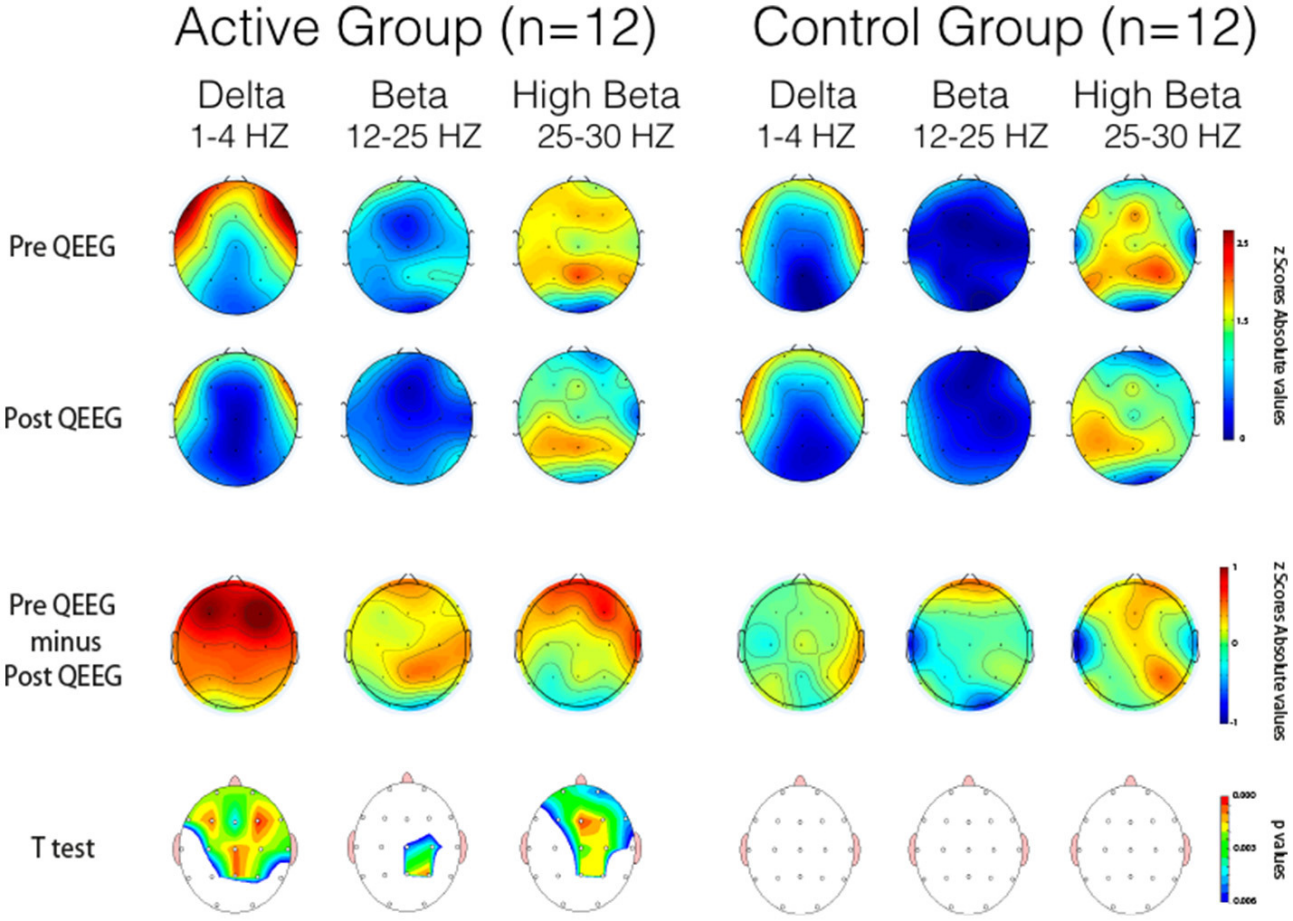
Pre-post qEEG Absolute Z Scores.

**Figure 3:**
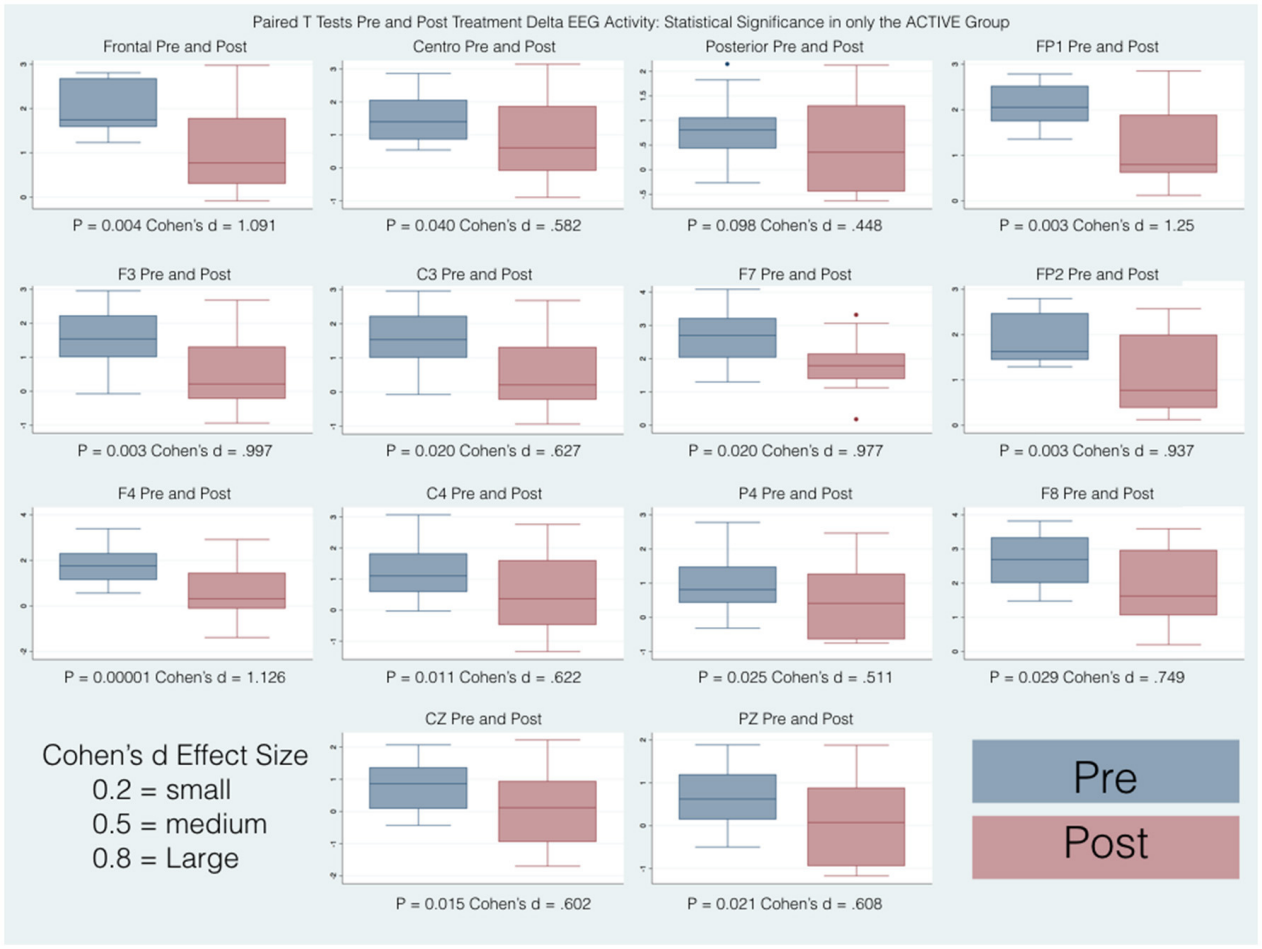
Box plots of statistically and substantively significant delta band widths pre and post treatment. Refer to tables for statistical and substantive significance.

**Figure 4:**
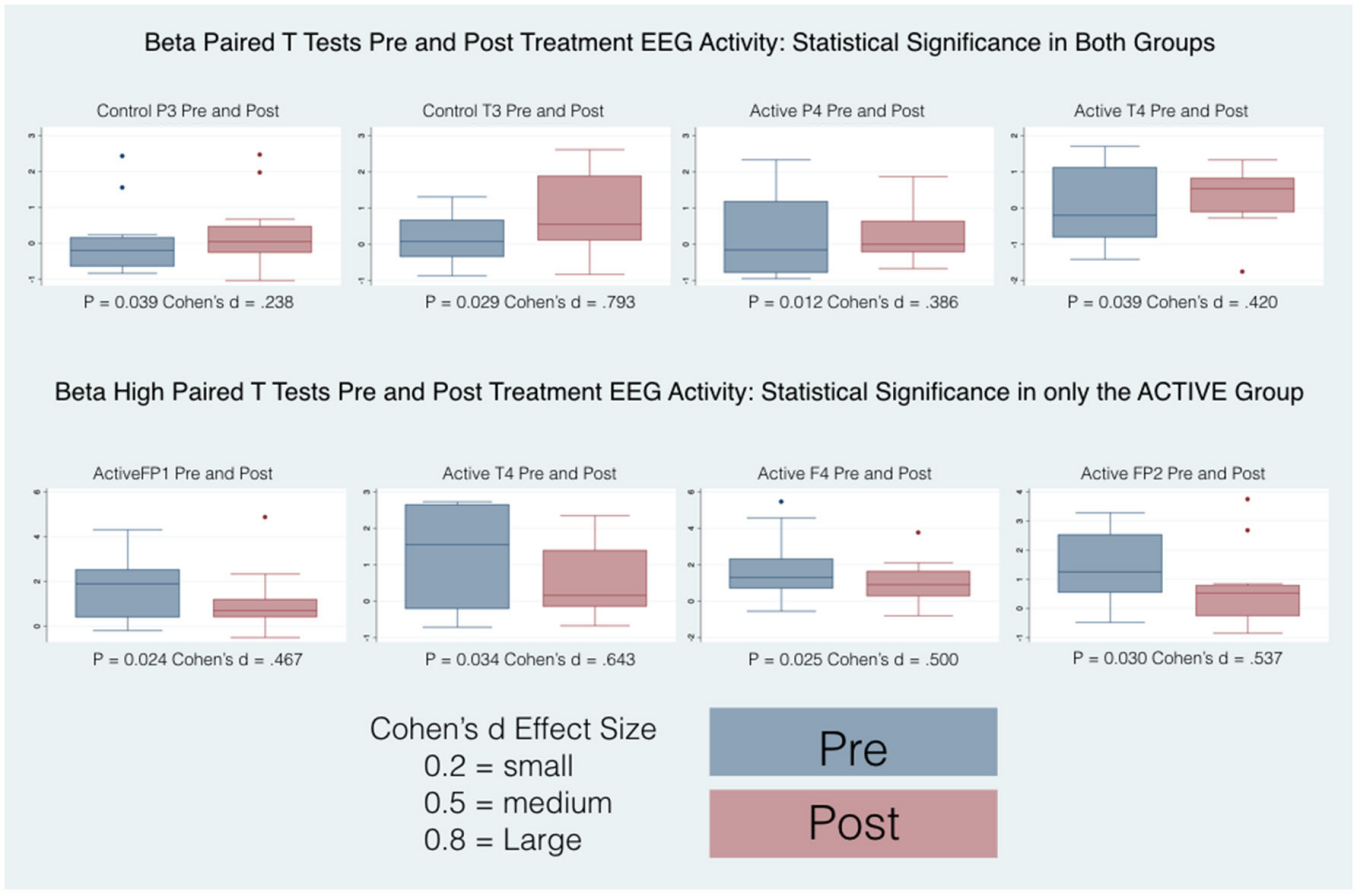
Box plots of statistically and substantively significant beta and high beta band widths pre and post treatment. Refer to tables for statistical and substantive significance.

Thirty-four participants completed the pre and post treatment qEEG testing. As stated before, because of artifact from movement and problems with tolerating the EEG cap, 10 subjects were removed from the analysis. There was some asymmetry in the qEEG results but this was not statistically significant. Three frequency bands demonstrated statistically significant changes pre and post treatment: delta (1-4 Hz), beta (12-25 Hz) and high beta (25-30 Hz). Our analysis reporting is specific for these areas and also focused on three main region of interest (ROIs): Frontal (Fp1, Fp2, F3, F4, F7, F8, Fz), Central (T3, T4, C3, C4, Cz), and Posterior (P3, P4, Pz, T5, T6, O1, O2,) that were chosen a priori for each frequency band to evaluate qEEG changes. The results are reported numerically in Table 2 and in graphical format in Figures 2–4.

**Table 2:** Numerical results of the statistical analysis performed on the qEEG Delta, Beta and High Beta bands.

**Variable**			**Frontal**			**Central**			**Posterior**		
**Group**			**Control**	**Active**	*p*-value^*^	**Control**	**Active**	*p*-value^*^	**Control**	**Active**	*p*-value^*^
Delta (1–4 Hz)	Pre	Mean (Std.Error)	1.325 (0.374)	1.977 (0.167)	0.126	1.119 (0.412)	1.511 (0.215)	0.408	0.474 (0.281)	0.420 (0.273)	0.273
	Post	Mean (Std.Error)	1.301 (0.363)	1.089 (0.287)		1.061 (0.403)	0.892 (0.377)		0.855 (0.190)	0.480 (0.285)	
	*p*-value^**^ Partial η^2^ Obs.-power		0.947	**0.003** **0.555** **0.920**		0.863	**0.040** **0.329** **0.563**		0.825	0.098	
Beta (12–25 Hz)	Pre	Mean (Std.Error)	0.520 (0.328)	0.874 (0.304)	0.438	0.201 (0.264)	0.743 (0.311)	0.197	0.367 (0.302)	0.786 (0.319)	0.351
	Post	Mean (Std.Error)	0.313 (0.214)	0.587 (0.308)		0.415 (0.185)	0.445 (0.251)		0.526 (0.237)	0.712 (0.265)	
	*p*-value^**^ Partial η^2^ Obs.-power		0.575	0.199		0.318	**0.031** **0.358** **0.617**		0.344	0.651	
High Beta (25–30 Hz)	Pre	Mean (Std.Error)	1.369 (0.355)	1.656 (0.431)	0.613	1.115 (0.267)	1.485 (0.414)	0.461	1.499 (0.308)	1.485 (0.388)	0.979
	Post	Mean (Std.Error)	1.088 (0.222)	1.107 (0.358)		1.352 (0.270)	1.159 (0.338)		1.348 (0.319)	1.549 (0.381)	
	*p*-value^**^ Partial η^2^ Obs.-power		0.431	**0.024** **0.382** **0.662**		0.342	0.146		0.621	0.810	

*When the p-value was significant (p < 0.05), the partial η^2^ (an estimation of the effect size: 0.02~small; 0.13~medium; 0.26~large) and the observed power (the probability of correctly rejecting the null hypothesis) are also reported*.

* *Assuming equal variances*.

** *Paired differences*.

*In bold the results that are statistically significant*.

Topographical statistical distribution of the outcomes after 12 weeks period of treatment in eyes closed condition assessed by comparing the Pre- and Post- qEEG separately for the Delta, Beta and High Beta bands, in the Active (left side) and the Control (right side) group respectively. Values are expressed in term of absolute Z scores and p values of the paired *t*-tests. With the reference to the t values the lower the values, the stronger the reductions of the abnormal Z scores values in the Post- over the Pre- qEEG assessment. Pre minus Post qEEG by Frequency Band demonstrates topographical statistical distribution of the outcomes after 12 weeks period of treatment in eyes closed condition assessed by comparing the Pre- and Post- qEEG separately for the Delta, Beta and High Beta bands. With the reference to the values on the respective color-coded bar the red indicates larger values on PRE and blue larger values on POST phase. With the reference to the absolute difference values on the respective color-coded bar the red indicates that the color is Red is higher and Blue is lower.

Pairwise comparisons within the groups (Active and Control), carried out for each band of frequency (Delta, Beta, High Beta), showed statistically significant results only in the Active group between pre and post scores. Overall changes represented an improvement toward normalization, i.e. a reduction of the abnormal values in the z-scores. In particular, the paired-samples *t*-test indicated that absolute z-scores were significantly lower (in bold in Table 2) in the post- versus pre-qEEG for the Active group in the Delta Frontal, Delta Central, Beta Central, and High Beta Frontal. Box plots comparing the statistically and substantively significant /Pre and Post results for the Delta, Beta and High Beta bands for the Active and Control groups are reported in Figures 3, 4.

### Posturography

No significant differences in the PRE and POST assessments between Active and Control groups nor in the Active and Control groups PRE-POST were found in the multivariate GLM analyzes. Table 3 contains for each of the considered posturography variables the average and standard error of the mean for each group pre and post treatment. The table shows horizontally the differences between Active and Control groups in the PRE and POST evaluations, and vertically the differences PRE-POST for the two groups separately. The results of the GLM analysis are also shown as the p value between groups pre and post and for each group pre-post. When the p value was significant (p < 0.05), the partial η^2^ (an estimation of the effect size: 0.02 ~ small; 0.13 ~ medium; 0.26 ~ large) and the observed power (the probability of correctly rejecting the null hypothesis) are also reported.

**Table 3:** Numerical results of the univariate GLM statistical analysis performed on the posturography data.

**Variable**			**95% Conf ML sway [mm/m]**			**95% Conf AP sway [mm/m]**			**95% Conf max sway [mm/m]**			**Average sway Vel [mm/s/m]**			**95% Conf ellipse Area [mm**^**∧2**^**/m**^**∧2**^**]**		
**Group**			**Control**	**Active**	***p*-value**	**Control**	**Active**	***p*-value**	**Control**	**Active**	***p*-value**	**Control**	**Active**	***p*-value**	**Control**	**Active**	***p*-value**
mCTSIB	Pre	Mean (Std.Error)	60.6 (11.6)	71.4 (13.0)	0.541	63.7 (12.8)	74.2 (15.2)	0.604	74.2 (14.4)	89.6 (17.4)	0.500	76.6 (18.3)	65.4 (13.2)	0.624	5037.4 (1788.9)	7004.0 (2401.1)	0.516
	Post	Mean (Std.Error)	53.9 (7.9)	48.7 (10.1)	0.686	51.4 (7.9)	47.4 (8.8)	0.733	64.1 (9.9)	57.9 (11.3)	0.680	53.0 (8.8)	46.3 (9.2)	0.605	3030.6 (920.8)	2804.6 (1000.1)	0.869
	*p*-value Partial η^2^ Obs.-power		0.404	**0.024** **0.281** **0.652**		0.210	**0.049** **0.220** **0.515**		0.299	**0.035** **0.248** **0.579**		0.093	0.081		0.169	**0.050** **0.219** **0.511**	
Hard surface eyes open	Pre	Mean (Std.Error)	61.1 (17.1)	85.2 (22.0)	0.394	59.2 (13.7)	88.6 (25.1)	0.311	70.7 (17.6)	112.7 (28.6)	0.221	77.2 (19.4)	62.1 (18.2)	0.575	5450.3 (2383.4)	10.443.7 (4815.2)	0.360
	Post	Mean (Std.Error)	59.5 (12.3)	42.4 (12.4)	0.324	56.6 (41.4)	41.4 (10.4)	0.330	*72.3* *(13.7)*	51.8 (14.6)	0.313	52.9 (11.0)	34.5 (8.6)	0.196	3782.0 (1272.2)	2218.2 (911.6)	0.325
	*p*-value Partial η^2^ Obs.-power		0.919	**0.013** **0.330** **0.750**		0.851	**0.043** **0.232** **0.542**		0.906	**0.015** **0.316** **0.724**		0.135	**0.039** **0.239** **0.559**		0.471	0.077	
Hard surface eyes close	Pre	Mean (Std.Error)	54.6 (13.5)	54.5 (14.5)	0.995	68.3 (19.1)	61.9 (14.5)	0.791	73.0 (19.2)	71.4 (16.2)	0.951	70.5 (25.1)	50.7 (13.5)	0.492	5725.9 (2526.4)	4680.2 (2437.0)	0.768
	Post	Mean (Std.Error)	51.4 (11.8)	44.3 (12.1)	0.676	46.5 (10.2)	47.0 (11.6)	0.977	59.0 (12.9)	57.2 (14.0)	0.925	51.4 (14.5)	48.6 (14.1)	0.891	3025.7 (1149.6)	2917.8 (1213.2)	0.949
	*p*-value Partial η^2^ Obs.-power		0.780	0.534		0.240	0.346		0.326	0.425		0.345	0.901		0.247	0.507	
Compliant surface eyes open	Pre	Mean (Std.Error)	53.7 (10.0)	66.7 (14.9)	0.474	59.0 (13.4)	60.0 (11.8)	0.953	69.7 (14.0)	75.8 (16.1)	0.778	75.0 (20.1)	60.5 (11.5)	0.535	3722.2 (1467.0)	4804.0 (1854.9)	0.650
	Post	Mean (Std.Error)	*56.6* *(8.7)*	45.5 (7.9)	0.351	51.3 (13.3)	43.0 (7.0)	0.582	67.9 (13.9)	51.4 (8.3)	0.316	55.6 (11.4)	41.7 (7.2)	0.311	3070.7 (1384.5)	2071.7 (550.8)	0.507
	*p*-value Partial η^2^ Obs.-power		0.762	0.107		0.416	**0.046** **0.227** **0.530**		0.863	0.070		0.193	0.088		0.499	0.103	
Compliant surface eyes closed	Pre	Mean (Std.Error)	72.9 (12.7)	79.1 (13.6)	0.742	68.4 (11.2)	86.1 (22.3)	0.483	83.4 (12.9)	98.4 (23.4)	0.577	83.7 (16.2)	88.4 (18.3)	0.849	5251.0 (1794.5)	8088.2 (3845.1)	0.509
	Post	Mean (Std.Error)	47.8 (6.2)	62.5 (12.2)	0.291	51.3 (6.2)	58.1 (9.4)	0.545	57.3 (6.9)	71.2 (12.4)	0.336	52.1 (7.1)	60.5 (14.0)	0.596	2243.9 (656.0)	4010.8 (1880.7)	0.382
	*p*-value Partial η^2^ Obs.-power		**0.026** **0.273** **0.634**	0.113		0.096	0.213		**0.033** **0.253** **0.589**	0.220		**0.036** **0.247** **0.575**	0.111		0.072	0.290	

*95% Conf MLSway = 95% confidence mediolateral sway, 95% Conf AP Sway = 95% confidence antero-posterior sway, 95% Conf Max Sway = the 95% confidence maximum sway (the largest sway in any direction), Average Sway Vel = average sway velocity (calculated as the sway path length (how much the CoP moved during the test) divided by the duration of the test), 95% Conf Ellipse Area = area of the 95% confidence ellipse*.

*In bold the results that are statistically significant, their partial η^2^ and observed power In underlined italic the results that got worse after treatment mCTSIB = all 4 posturography tests combined as repeated measures. In bold the results that are statistically significant. In underlined italic the results that are worse POST vs. PRE mCTSIB*.

For each subject, the different sway measurements and the average sway velocity were normalized by the subject's height, and the 95% confidence ellipse area was normalized by the square of the subject's height to remove any subject's gender and height dependency. When the p value was significant (p < 0.05), the partial η^2^ (an estimation of the effect size: 0.02 ~ small; 0.13 ~ medium; 0.26 ~ large) and the observed power (the probability of correctly rejecting the null hypothesis) are also reported.

**Figure 5:**
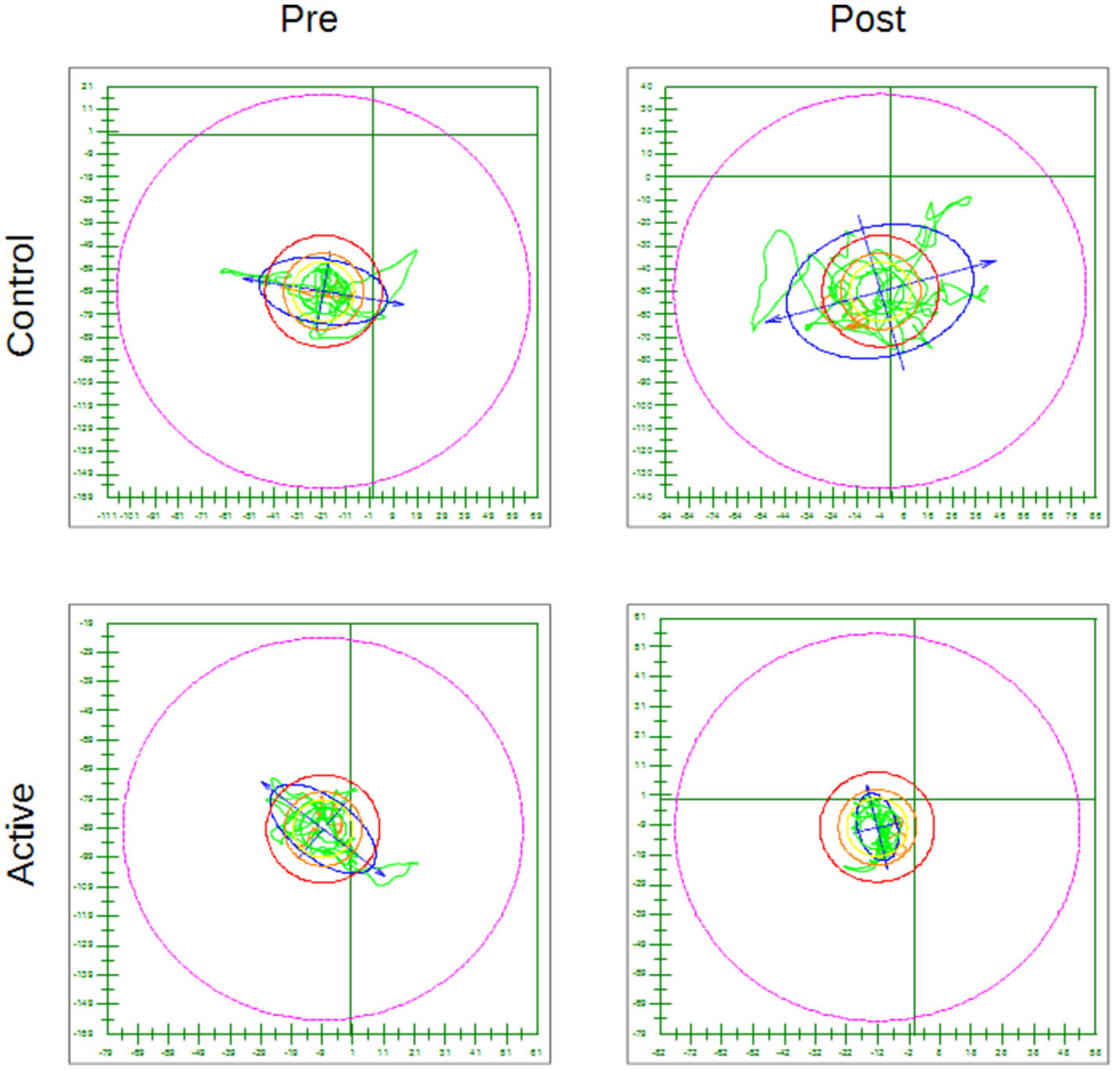
Examples of CoP trace for the hard surface eyes open condition pre and post treatment for a subject in the Control group and for one in the Active group—The outer circle represents 100% of the theoretical limit of stability for the individual subject. Everything else is scaled accordingly.

As an example Figure 5 shows for the hard surface eyes open test condition, the CoP trace pre and post treatment for a subject in the Active group (with a statistically significant decrease) and for one in the Control group (with an increase in whole body sway post placebo treatment).

### Questionnaires

**Figure 6:**
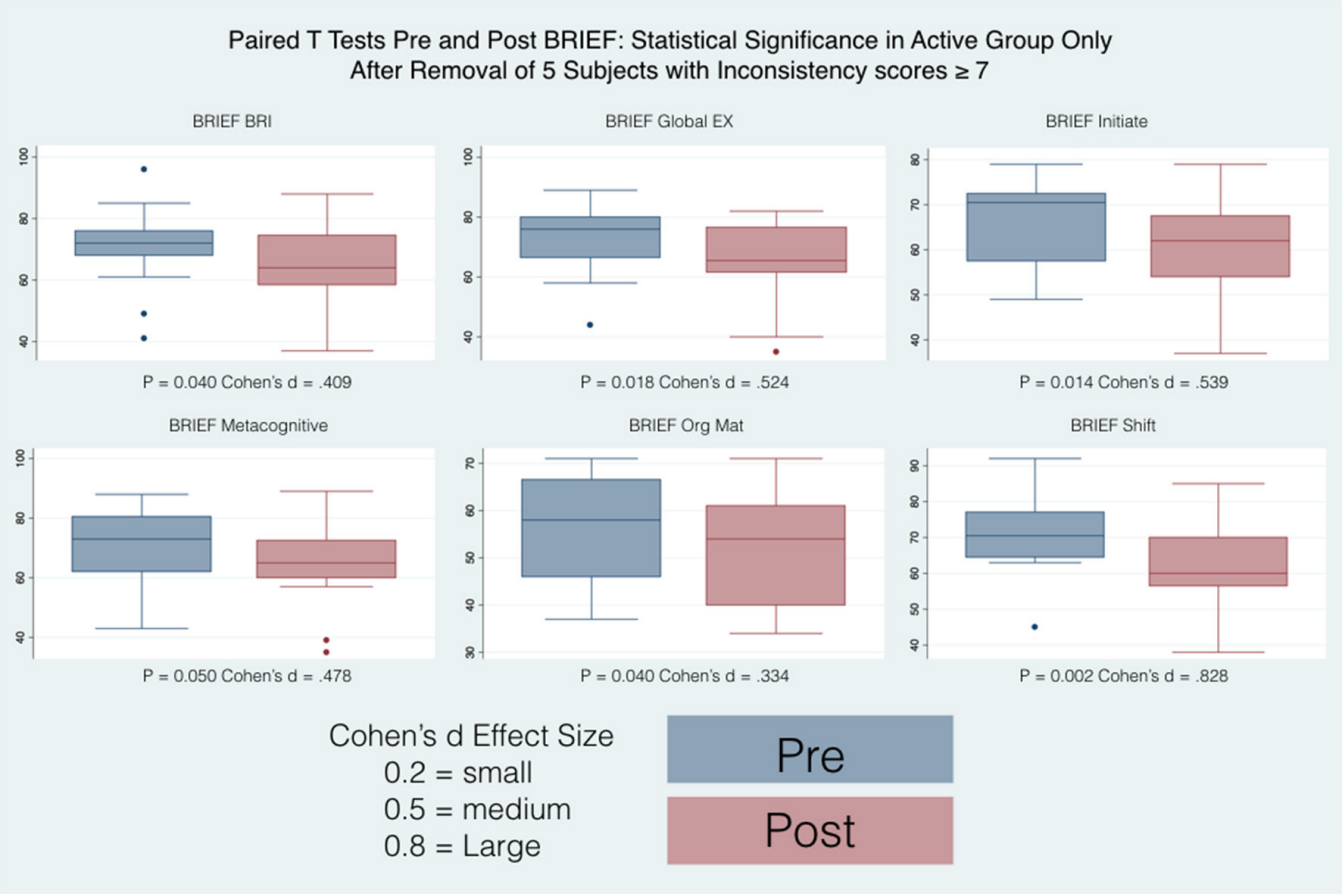
Box plots PRE and Post BRIEF after excluding subjects with high Inconsistency scale.

**Figure 7:**
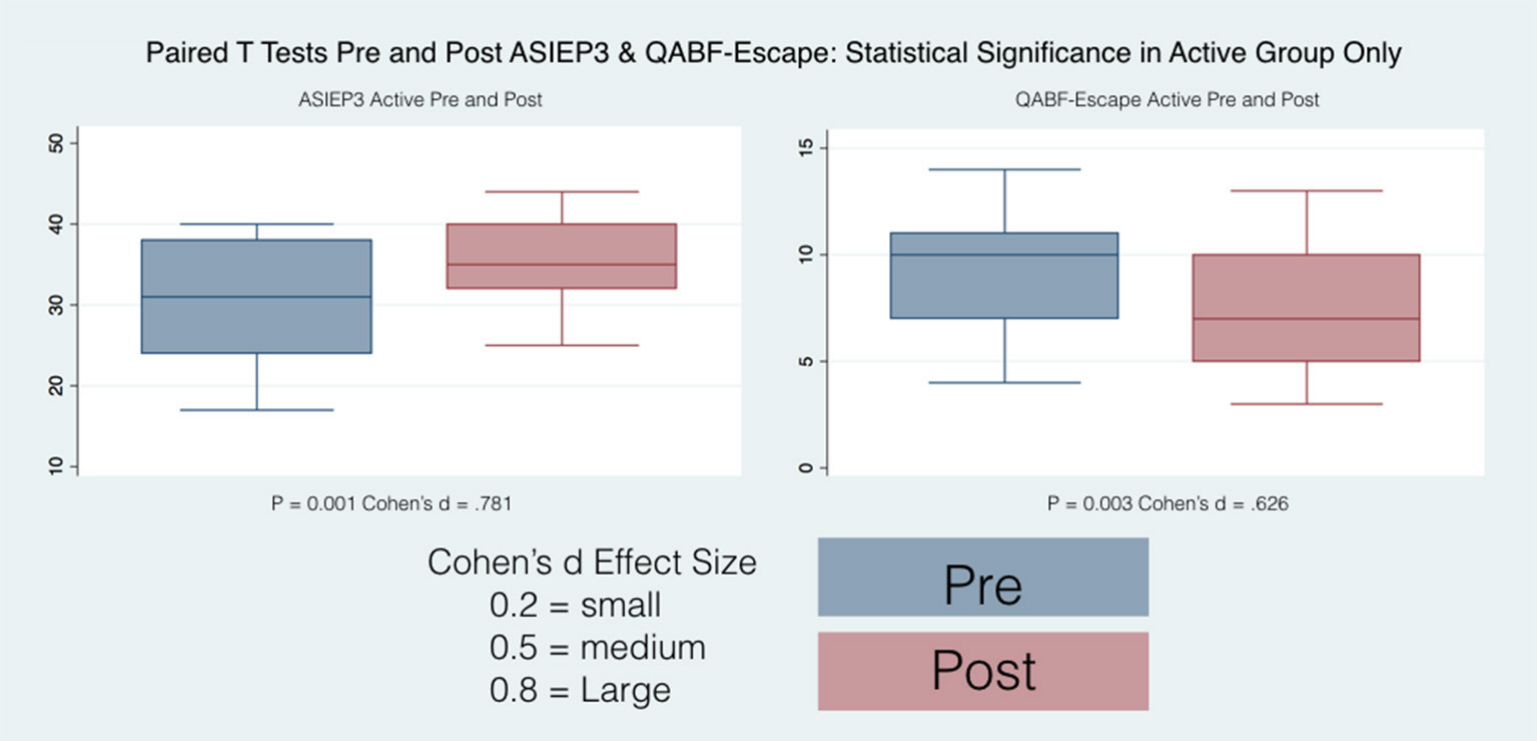
Box plots PRE and Post ADIEP3 and QABF Escape.

34 subjects (28 males and 6 females), completed the questionnaires at the beginning of the study and at their final outcome visit. We tested for differences in gender and only found differences between boys and girls at their starting point with reference to the Brief (Organizational Materials Subscale), ATEC (Speech) and SRS (2-Awareness). The T-scores obtained were adjusted for age and sex allowing us to combine all subjects independent of gender for our analysis. All the t values that are positive indicate an improvement in symptoms or dysfunctional areas according to the T-scores provided by each questionnaire standardization with the exception of the ATEC that has negative values indicating a worsening of symptoms. We included a Global Executive Composite (GEC), representing a summary score that incorporated all of the BRIEF clinical scales. Paired *t*-tests were calculated to explore the differences between pre- and post-test scores within the groups (Active andC), with regard to: standardized T-scores and for BRIEF-2, SRS-2, ABC (ASIEP-3): raw scores for QABF and ATEC. For each paired *t*-tests, an effect size was calculated as Cohen's d (21). The results of this statistical analysis are reported in Table 4 and the box plots for only the statistically significant results are reported in Figures 6, 7.

**Table 4:** Pre and Post questionnaire mean and standard error with bold significant values (^*^Assuming equal variances ^**^after excluding subjects with high Inconsistency scale).

**Questionnaire**		**SRS-2**																	
**Variable**		**Social awareness**			**Social cognition**			**Social communication**			**Social motivation**			**RRB**			**SCI**		
**Group**		**Control**	**Active**	p*	**Control**	**Active**	p*	**Control**	**Active**	p*	**Control**	**Active**	p*	**Control**	**Active**	p*	**Control**	**Active**	p*
Pre	Mean (Std.Err)	75.59 (2.585)	76.12 (2.588)	**0.034**	74.53 (1.851	72.18 (2.530)	0.223	76.94 (2.200)	76.41 (2.971)	0.507	73.18 (2.686)	70.41 (2.244)	0.251	81.88 (2.488)	79.29 (2.533)	0.782	78.18 (1.879)	76.82 (2.149)	0.127
Post	Mean (Std.Err)	*76.18* *(1.399)*	72.47 (2.017)		71.24 (1.917)	70.53 (2.330)		73.71 (2.774)	70.41 (2.244)		70.76 (2.258)	68.18 (2.403)		77.47 (3.131)	75.29 (2.533)		75.35 (1.788)	73.82 (2.459)	
	*p*-value Partial η^2^ Obs.P	0.846	0.206		0.144	0.525		0.072	0.161		0.182	0.352		0.079	0.174		0.106	0.166	
**Questionnaire**		**ABC**			**ATEC**														
**Variable**		**Total raw score**			**Speech / Language communication**			**Sociability**			**Sensory / Cognitive awareness**			**Health / Physical behavior**			**Total**		
**Group**		**Control**	**Active**	p*	**Control**	**Active**	p*	**Control**	**Active**	p*	**Control**	**Active**	p*	**Control**	**Active**	p*	**Control**	**Active**	
Pre	Mean (Std.Err)	85.76 (350)	89.41 (3.239)	0.462	17.00 (1.683)	18.59 (1.787)	**0.014**	12.29 (1.686)	14.59 (1.665)	0.639	21.29 (1.233)	20.12 (1.981)	0.122	23.65 (3.280)	23.24 (2.289)	0.276	74.24 (4.96)	76.53 (3.68)	
Post	Mean (Std.Err)	85.12 (2.484)	80.59 (2.773)		*18.35* *(1.372)*	*19.65* *(1.855)*		11.00 (1.663)	10.59 (1.412)		*22.88* *(1.450)*	*22.53* *(2.220)*		19.76 (3.095)	19.88 (2.009)		72.00 (4.10)	72.65 (3.37)	
	*p*-value Partial η^2^ Obs.P	0.816	**0.002 0.460 0.933**		0.057	**0.006 0.381 0.838**		0.217	**0.022 0.288 0.665**		0.244	**0.050 0.219 0.513**		**0.004 0.411 0.880**	0.071		0.233	0.164	
**Questionnaire**		**QAFB**																	
**Variable**		**Social attention**			**Escape**			**Tangible reinforcement**			**Physical siscomfort**			**Nonsocial rehinforcement**			**Total**		
**Group**		**Control**	**Active**	p*	**Control**	**Active**	p*	**Control**	**Active**	p*	**Control**	**Active**	p*	**Control**	**Active**	p*	**Control**	**Active**	
Pre	Mean (Std.Err)	5.00 (0.804)	2.82 (0.671)	0.720	7.82 (1.075)	9.24 (0.730)	0.094	8.29 1.067)	9.24 (0.881)	0.128	5.94 (0.976)	6.65 (1.000)	0.309	8.47 (0.963)	9.94 (0.929)	0.245	35.53 (3.96)	40.88 (2.44)	
Post	Mean (Std.Err)	4.65 (0.747)	*5.47* *(0.697)*		*7.88* *(0.652)*	7.35 (0.727)		7.88 (0.861)	8.00 (1.085)		4.84 (0.656)	5.41 (0.936)		*9.06* *(0.972)*	8.24 (0.851)		34.41 (2.59)	34.47 (2.98)	
	*p*-value Partial η^2^ Obs.P	0.455	0.543		0.946	**0.003 0.424 0.896**		0.563	0.106		0.329	0.268		0.507	**0.035 0.249 0.580**		0.708	**0.043 0.232 0.542**	
**Questionnaire**		BRIEF**																	
**Variable**		**Inhibit**			**Shift**			**Emotional control**			**Behavioral regulation index**			**Initiate**			**Working memory**		
**Group**		**Control**	**Active**	p^*^	**Control**	**Active**	p^*^	**Control**	**Active**	p^*^	**Control**	**Active**	p^*^	**Control**	**Active**	p^*^	**Control**	**Active**	p^*^
Pre	Mean (Std.Err)	64.54 (3.155)	68.88 (3.551)	0.969	66.38 (3.666)	71.94 (2.823)	0.394	60.46 (3.854)	64.25 (3.406)	0.056	65.54 (3.762)	70.75 (3.241)	0.234	64.08 (2.971)	66.56 (2.420)	0.326	68.62 (2.571)	72.50 (2.941)	0.950
Post	Mean (Std.Err)	64.54 (3.155)	66.94 (3.740)		63.08 (3.917)	62.38 (2.947)		57.85 (3.561)	59.63 (3.275)		63.92 (2.863)	65.25 (3.483)		63.23 (2.585)	60.63 (3.049)		66.31 (2.929)	66.69 (3.127)	
	*p*-value Partial η^2^ Obs.P	1.000	0.521		0.185	**0.002 0.490 0.943**		0.164	0.080		0.391	**0.040 0.252 0.557**		0.546	**0.014 0.341 0.741**		0.278	0.059	
**Variable**		**Plan/Organize**			**Organization of materials**			**Monitor**			**Metagognition index**			**Global executive composite**					
**Group**		**Control**	**Active**	p*	**Control**	**Active**	p*	**Control**	**Active**	p*	**Control**	**Active**	p*	**Control**	**Active**	p*			
Pre	Mean (Std.Err)	66.46 (2.382)	70.88 (3.877)	0.207	53.85 (3.322)	56.00 (2.805)	**0.009**	65.46 (2.688)	71.31 (2.755)	0.101	66.08 (2.302)	70.75 (3.041)	0.572	67.31 (2.863)	72.19 (2.830)	0.735			
Post	Mean (Std.Err)	61.85 (3.048)	65.94 (3.416)		52.62 (3.079)	52.13 (3.000)		65.15 (3.658)	64.81 (3.639)		63.77 (2.920)	64.56 (3.417)		64.92 (3.294)	65.75 (3.290)				
	*p*-value Partial η^2^ Obs.P	0.164	0.158		0.473	**0.040 0.253 0.559**		0.937	0.056		0.245	**0.050 0.232 0.513**		0.217	**0.018 0.322 0.704**				

*In bold the results that are statistically significant. In underlined italic the results that are worse POST vs. PRE mCTSIB*.

#### The autism treatment evaluation checklist (ATEC)

Statistically significant results were found in both groups but with different directions of the effects for the single scales: For the Active group, the behavior worsened for the Speech and the Sensory/Cognitive Awareness whereas the Sociability improved. For the Control group, only the Health/Physical Behavior showed a statistically significant improvement No statistically significant results were found with the reference to the Total score.

#### The social responsiveness scale–second edition (SRS-2)

As mentioned previously, T- scores were transformed from the raw individual scores thus allowing a comparison for age and gender. No statistically significant results were found with the reference to each clinical scale as well as the two composite indexes, RRB and SCI for either the Active or Control groups.

#### The behavior rating inventory of executive function (BRIEF)

T-scores were transformed from the raw individual scores thus allowing the comparison for age and gender. Among significant results, the direction of the effects pointed toward an improvement of executive function where high T scores indicated clinically relevant observations with the reference to each functional area as described by the individual subscales. When all the subjects are considered, The Active group shows significant differences between pre and post only in the Shift subscale whereas the Control group shows significant differences between pre and post in the Shift, Plan/Organize, Metacognition Index and in the Global Executive Composite. When the subjects with high inconsistency are excluded from the analysis (Table 4), then only the Active group show significant differences pre and post, in particular in the Shift, Initiate, Organizational of Materials, Behavioral Regulation Index, Metacognition Index, and in the Global Executive Composite.

#### The autism behavior checklist (ABC)

Only the Active group had statistically significant differences between pre and post scores and the direction of the effect was toward a reduction of the autistic behaviors.

#### Questions about behavioral function (QABF)

Only the Active group had statistically significant differences between pre and post scores for the Escape, the Nonsocial Reinforcement and the total score. The direction of the effect was toward a reduction of problematic behaviors.

### Qualitative reporting

Parents of subjects in the Active group of this investigation reported significant improvements in communication and social skills of their children while the parents of subjects in the Control group did not report much change in these skills.

## Discussion

The Mente device represents a novel approach in a NFB approach to ASD treatment. There are no other comparisons represented in the literature. As shown in Table 1 (demographics for the posturography analysis) and in Tables 2–4 for the PRE evaluation using the qEEG, posturography and questionnaires, no significant differences were found between the Active and the Control groups with the exception of the Social Awareness score of the SRS-2 questionnaire, the Speech / Language Communication of the ATEC and the Organization of Materials of the BRIEF (in bold in Table 4), so whatever difference were found between PRE and POST treatment evaluations was most likely due to the treatment itself, rather than the subjects. We realized that qEEG is an essential tool for the evaluation and treatment of neurophysiologic disorders (31) but had major difficulties with many of our subjects not tolerating the EEG cap on their heads or having behavioral issues that prevented the acquisition of EEG without artifact and as we discussed previously, 24 subjects could be included in the qEEG analysis (Table 1). In spite of these difficulties, our main findings represent statistically significant improvements of Delta, Beta and High Beta Frequencies in the Active group after a 12 weeks NFB home treatment with the Mente device.

The various frequency bands of the EEG represent power spectra that are regulated by anatomically homeostatic systems mediated by cortical, thalamic and brainstem processes (32). Changes in the power spectra of the EEG as observed in this investigation reflect a statistically significant change in brain function of the active treatment group as a consequence of the intervention. Studies using qEEG in ASD have shown that there is significant lower spectrogram criteria values in left brain hemisphere at F3 and T3 electrodes and at FP1, F7, C3, Cz and T5 electrodes (33). We observed similar criteria values in the active treatment group with subsequent improvement after treatment. We have documented non significant brain symmetry differences in all of our subjects (Active and Control) before treatment with subsequent changes after treatment. Abnormal functional brain lateralization has been reported before in EEG recordings of ASD subjects (34). Low EEG frequencies tend to decrease with age from childhood to adulthood while high frequencies increase (35). We observed changes in the qEEG frequencies with noted decreases of low EEG frequencies and increases of high frequencies in our Active group after treatment signifying a trend toward normality. For the most part we did not find statistically significant changes in our Control group after treatment but did find a statistically significant improvement only in the Beta band P3 and T3 leads.

Sensory-based interventions (SBIs) are associated with an improved performance in daily life activities and occupations of people with ASD (36) and although the sham treatment did not affect significant change in general, it does appear that the sham sound had a minor consequence in brain activity. Sensory stimulation such as sound may be modified by environmental factors in the development of an individual whose brain interacts with the environment with resultant modification of neural circuitry (37). An assessment of Cochrane systematic reviews found that music therapy is associated with evidence of benefits for patients with autism spectrum disorders, although the evidence is low quality with a need for high quality long term clinical trials (38). The presentation of binaural auditory beats can affect psychomotor performance and mood, comprising a dual complex system including spectral complexities that are effective for ASD children (15). The binaural beats associated with the NFB of the Active Mente device are associated with the differences between the Active and Control groups. Spectral complexity is present due to different frequencies being sent back to the user in the Active group's NFB loop, resulting in the generation of EEG data frequencies. Binaural beats originate in the superior olivary nucleus as a new auditory stimulation produced by listening to different frequencies of sound at each ear (39). Our investigation demonstrated significant changes in the qEEG of various brain areas and no significant changes in others. The cortical processing evoked by binaural beats is similar in distribution to other acoustic beats that are located mostly to left temporal lobe areas (40). However, multisensory temporal integration in ASD is associated with a wider temporal binding window suggesting that general sound stimulation may bind with other sensory modalities such as proprioception of the head band resulting in an improvement in postural stability (41).

Considering the posturography results (Table 3 and Figure 5) some of the changes recorded between PRE and POST were very small (such as in the case of the 95% Conf. ML sway for the Control group in the hard surface eyes open and closed tests and of the 95% Conf. Max Sway again for the Control group in the eyes open on hard or compliant surface tests) and might have been gone undetected if the instrument used to record the posturography data were not sensitive enough. Looking at the results in general, we found a decrease in whole body sway in both the Active and Control groups but only the Active group demonstrated statistically significant decreases in sway after treatment. The proprioceptive effect of the headband itself might contribute to central changes independent of sound and should be investigated. Although each posturography test lasted 20 seconds, as shown in Table 1, only 34 of the enrolled subjects had a full set of posturography evaluations pre and post treatment and were included in the analysis. This was mainly due to the difficulty in keeping the child in position for the entire duration of the test and obtaining meaningful data without excessive movements or the subject stepping out of position, or opening the eyes. Some subjects were even jumping up and down on the CAPS force platform, but fortunately this type of abuse did not injure the children nor damage the instrument, because of a designed maximum load of 1,000 kg. Also the larger area of support allowed subjects to assume their preferred stance without any restrictions. It would be expected that the more difficult the test, the higher the sway would be. However, this was not always the case: for example the 95% Conf ML sway in the hard surface eyes open results were actually worse than the corresponding compliant surface eyes closed results pre treatment for the Active group (85.2 mm/m pre vs. 79.1 mm/m post) or the 95% conf ellipse area in the hard surface eyes open results were also worse than the corresponding compliant surface eyes open results pre treatment for the Control group (5450 mm^2^/m^2^ vs. 3722.2 mm^2^/m^2^).

It is expected that people are able to stand better when they can see their environment and these findings in our ASD subjects are alarming. ASD individuals experience the world around them uniquely by using different visual strategies to process social information (42). Clearly, the comparison of the performance of vision strategies and eye movements and gaze following in social settings is quantitatively different in children with developmental delays compared to children without delays (43). Obviously, the role of the eyes in gaze holding plays a central role in social cognition that is impaired in ASD children, suggesting a deficiency in the spatiotemporal networks of the brain (44). The worsening of stability and sway with the eyes open in our subjects may be identified as a biomarker for ASD. Even the targets that a child looks at before maintaining gaze are different in ASD than typically developing children who look at the eyes and mouth of an individual much more than an autistic child (45). A recent meta-analysis suggests that decreased eye fixation to the eye region of the face may represent a robust biomarker for ASD (46). Interestingly, there are differences in fixation time at eye regions between ASD children who tend to fixate on the right eye for a greater amount of time than the left eye compared to typically developing children that increase left eye fixation over right eye fixation when scanning the face (47). Individuals with autism have difficulty in following another person's gaze direction suggesting pathology linking the orbitofrontal cortex with the superior temporal sulcus and amygdala (48) confirmed by combining eye tracking and functional neuroimaging (49). In order to learn, infants must share joint attention with others and be able to follow their gaze toward goal objects (50), something that ASD children do poorly. Autistic children also have difficulty encoding features of another's face, gaze and facial movements while at the same time demonstrating an inability to imitate body actions (51). These measurable performances are found in concert with difficulties of executive function, memory and language abilities and social sensitivity with a lack of eye gaze alternation and socialization of ASD children compared to typically developing youths (52).

The increased ML sway probably represents a greater frequency of moving weight from one side to the other representing an increase of fidgety movements in the Control group. When comparing pre and post treatment results, on average, with the exception (in red in Table 2) of the 95% Conf. ML sway for the Control group in the compliant surface eyes open test and the 95% Conf. Max sway for the Control group in the hard surface eyes open test, all average values improved (became smaller) for both the Active and the Control groups between pre and post treatment, indicating that even if not in synch with the EEG the sound stimulation had an effect (53). However, this effect was statistically significant only for the 95% confidence measures when the mCTSIB results were considered. Similar results were obtained when considering the hard surface eyes open test with the 95% confidence sway measures and the average sway velocity again all with statistically significant differences between pre and post for the Active group. For the Active group, no statistically significant differences were found for the other three tests comprising the mCTSIB, with the exception of the 95% Conf. AP Sway. For the Control group most variables across all tests decreased in value, but these changes where statistically significant for the 95% Conf. ML Sway, the 95% Conf. Max Sway and the average sway velocity of the PSEC test. This explains the changes in the Control group posturographic tests and further emphasizes the benefit of specific brain based sound stimulation as demonstrated in the statistically significant improvements in the group in this study. These were also confirmed by the results obtained from the statistical analysis of the questionnaires results (Table 4 and Figures 6, 7).

Furthermore, the emotional impairments in ASD may be broader than just a mere consequence of social impairments (54) that can be problematic for developing children especially when complicated by aberrant signaling and perception of internal bodily sensation referred to as interoception. The atypical interoception observed in ASD is thought to be due to a pathological processing of multisensory connections and integration in cortical and subcortical areas (55). It is reasonable to suggest that aberrant sensorimotor integration with feed forward pathophysiology is associated with the motor impairments and motor control disturbances commonly observed in ASD (56). Such disturbances are associated with a persisting impairment of maintaining postural control that begins in infancy and that might serve as an early diagnostic marker or endophenotype of autism (57). These impairments are associated with patterns of postural control and memory performance-attention deficits that are associated with emotional language processing in ASD (54). The relationships of postural control mechanisms and cognitive functions suggest that both should be utilized as measurements in the diagnosis and treatment of ASD patients. For instance, abnormalities in the development of spontaneous fidgety movement patterns of infancy are seen in ASD and are associated with deficits in language skills and cognition at school age (58). Besides these deficits, children that are not able to stand still or constantly shift their weight from leg to leg will be expected to show differences in their center of pressure (CoP) that might be measured by posturography. The CoP measured by posturography involves a trajectory occurring from a shift in the body weight from one foot to the other during quiet stance allowing for identification of stable and unstable postural control (59). The measurement of CoP by posturography may also identify biomarkers of ASD as postural control systems are problematic, starting in infancy and continuing throughout the lifespan (57). ASD infants demonstrate a motor pathology of postural control as their heads may characteristically drop backwards when they are held horizontally (1). Interestingly, in spite of well documented motor pathology in ASD, the diagnostic criteria of the spectrum does not typically include motor symptomatology (60). It is known that ASD is associated with aberrancies in sensorimotor processing such that postural instability suffers when visual feedback is utilized over somatosensory information (61). Furthermore, pathology in the motor fronto-striatal and cerebellar systems are associated with difficulty of cognitive functions and movement control associated with walking, posture and ataxia similar to that seen in ASD (62).

Parents of subjects in the Active group of this investigation reported significant improvements in communication and social skills as have other investigators of the effects of neurofeedback treatment in ASD (63). These were also confirmed by the results obtained from the statistical analysis of the questionnaires results (Table 4 and Figures 6, 7). Only the SRS-2 questionnaire did not show any statistically significant change although all its scores showed a decrease in value (an improvement) for both the Active and the Control groups with the exception of the Social Awareness in the Control group (in underline italic in Table 4). Not all the indexes showed an improvement: for both the Active and Control group the Speech / Language Communication and the Sensory/ Cognitive Awareness of the ATEC had an increase in value (statistically significant in the Active group - in underline italic in Table 4) indicating a worsening between PRE and Post evaluations. Similarly, the Active group showed a worsening in the Social Attention score, and the Control group in the Escape and Nonsocial Reinforcement in the QAFB questionnaires (although not statistically significant - again in underline italic in Table 4). The implications of this need to be considered with utilization of the Mente device with further investigation in these areas needed. The Control group showed a statistically significant improvement only in the Health/Physical Behavior of the ATEC questionnaire, whereas the Active group had statistically significant improvements in the scores of the ABC questionnaire, of the Sociability scale of the ATEC questionnaire, of the Escape, Nonsocial Reinforcement and Total scores of the QAFB questionnaire, and of the Shift, BRI, Initiate, Organization of Materials, MCI and GEC of the BRIEF questionnaire. Clearly, some of the questionnaires demonstrated significant changes whereas others did not indicating that the questionnaires are all specific for different measurements as we have detailed. As the questionnaires are validated instruments utilized in clinical applications around the globe, the significant positive changes in the Active group compared to the Control group are supportive of the positive effects of the treatment and are in concert with the physical measurements we have reported.

## Limitations

There were several limitations in this investigation. The large percentage of drops outs due to an inability or lack of importance to return for a post treatment examination demanded the randomization of a larger number of subjects to compensate and obtain the sample size planned for and the results may be biased by the drop-out rate. No subjects dropped out because of problems of tolerance with the Mente device or treatment. Although the parents of the Active group were generally pleased with the treatment outcomes, there were a large number that did not embrace the need to return for outcome studies due to costs of their visits. We feel that future studies providing the cost of transportation and lodging for subjects would contribute to better compliance with outcome testing but may be beyond funding reality. The physical and emotional characteristics of ASD children do make standardized testing a challenge. They find it difficult to stand on platforms, tolerate electrode caps and following instructions. Furthermore, this investigation was conducted at one neurological center and the results might not be generalized to a larger population. Most treatments of ASD involve multiple therapies at the same times and the isolation of an individual therapy that might act better in concert or combination with other therapies is not realized in this study. We used qEEG as an outcome measurement in this study and found it to be a robust tool. However, throughout the literature, alterations in the power of specific frequency bands in qEEG have been described in ASD, but they are very far from being a diagnostic test that is used in common clinical practice. Neurofeedback is a promising way to help children with ASD but some typical limitations of such technique should be taken into consideration. Conventional neurofeedback treatments are usually offered by professionals in their offices. This implies a considerable effort from parents, and patients themselves, both in terms of time and resources. Doing the therapy in clinical environments may sometimes increase the patients' stress and could affect the therapy outcomes. Along with the limitations of the traditional neurofeedback approach, there is the limited number of sessions that professionals can offer, and the patient can sustain (one or two per week), due to the transfers home-office and the cost of the session. Another limitation is that EEG is usually collected by means of caps (or single electrodes) to be placed on the scalp and wet with a sticky electroconductive gel. Also, the EEG cap usually comes with wires that can limit the patient's movements. Depending on the context and the severity of symptoms, this may cause discomfort to ASD patients that often don't like to be touched or constrained. Thus, a wireless device equipped with dry sensors and specifically designed for home-based treatment as in this study is of benefit and thought to complement traditional NFB therapies is highly desirable and easy to use for not trained people (i.e. parents).

## Conclusions

In conclusion, the outcomes of this investigation suggest that the use of the Mente Autism device as a novel NFB tool is associated with significant positive changes in the neuroregulation of ASD children. This 12 weeks home based randomized placebo controlled trial resulted in statistically significant changes in qEEG, Posturography and standard questionnaires used in the assessment of children affected by ASD. The changes were associated with statistically and substantively significant changes for subjects that received the treatment and not for those in the placebo controlled group. The use of the Mente Autism device shows promise as an addition to a treatment program of ASD. Furthermore, our results confirmed reports in the literature that qEEG is able to detect changes produced by NBF in children affected by ASD. The utilization of posturography as an alternative method to detect such changes is beneficial but demands that the instrument be sensitive enough to detect small changes and sturdy and robust enough to take the abuse some of the ASD children inflict to the devices during data collection. Mente Autism has a unique approach to target ASD using a home-based NFB treatment in an easy way for patients and parents. It can represent a valid complementary tool to increase the effectiveness of traditional NFB therapies since part of the therapy can be carried on at home, every day, ensuring continuity and comfort for patients and their families.

## Presentations

The preliminary findings of this study were presented at the 6th Cambridge International Conference on Mental Health at Claire College, University of Cambridge, Cambridge, UK held on September 20–22, 2017 and at the International Symposium for Clinical Neuroscience, Orlando, Florida, USA held on May 24-26, 2018.

## Disclosures

GP and EO are affiliated with and have financial interests in Vestibular Technologies, LLC, the company that manufactures and sells the CAPS Professional System used to obtain posturographic data in this study. None of the other authors have any disclosures or conflicts of interest that might affect this investigation.

## Author contributions

FC, GP, AH, MA, RZ, EK, DB, PL, and EO contributed to the conception and design of the study. EK, DB, and PL organized the database. FC and GP performed the statistical analysis. FC wrote the first draft of the manuscript. GP, AH, MA, RZ, EK, DB, PL, and EO wrote sections of the manuscript. All authors contributed to manuscript revision, read and approved the submitted version.

### Conflict of interest statement

The authors declare that the research was conducted in the absence of any commercial or financial relationships that could be construed as a potential conflict of interest.
